# The First Identification of *Cryptosporidium parvum* Virus-1 (CSpV1) in Hanwoo (*Bos taurus coreanae*) Calves in Korea

**DOI:** 10.3390/vetsci10110633

**Published:** 2023-10-26

**Authors:** Jeong-Byoung Chae, Seung-Uk Shin, Serim Kim, Young-Mi Jo, Hyunsoo Roh, Hansong Chae, Won-Gyeong Kim, Joon-Seok Chae, Hyuk Song, Jung-Won Kang

**Affiliations:** 1 Bio Team, Animal Industry Data Korea, Seoul 06152, Republic of Korea; cjb117@snu.ac.kr (J.-B.C.); lucas@aidkr.com (S.-U.S.); semmy@aidkr.com (S.K.); joanne@aidkr.com (Y.-M.J.); levi@aidkr.com (H.R.); chloe.chae@aidkr.com (H.C.); kasey@aidkr.com (W.-G.K.); 2 Laboratory of Veterinary Internal Medicine, BK21 FOUR Future Veterinary Medicine Leading Education and Research Centre, Research Institute for Veterinary Science and College of Veterinary Medicine, Seoul 08826, Republic of Korea; jschae@snu.ac.kr; 3 Department of Stem Cells and Regenerative Technology, Konkuk Institute of Science and Technology, Konkuk University, Seoul 05029, Republic of Korea; songh@konkuk.ac.kr

**Keywords:** *Cryptosporidium parvum*, *Cryptosporidium parvum* virus-1, Hanwoo, prevalence, phylogenetic analysis

## Abstract

**Simple Summary:**

*Cryptosporidium* is a parasite that causes digestive diseases in cows, and a virus called *Cryptosporidium parvum* virus-1 (CSpV1) is associated with it. We investigated the presence and features of CSpV1 in diarrhea samples of Hanwoo calves (Korean indigenous cattle). Out of 140 samples, 70 were positive for *Cryptosporidium* and 70 were negative. CSpV1 was found in 20% of the samples, more commonly in those already positive for the parasite. This is the first study on CSpV1 in Korea, offering insights into the relationship between the parasite and virus in cows.

**Abstract:**

*Cryptosporidium* is an obligate coccidian parasite that causes enteric diseases in bovine species. A double-stranded RNA virus associated with *C. parvum* oocysts, *Cryptosporidium parvum* virus-1 (CSpV1), has been characterized. However, the relationship between the abovementioned coccidian parasite and the virus has not been studied in the context of the known clinical outcomes. This study aimed to characterize the prevalence and molecular traits of CSpV1 in diarrheal feces of Hanwoo (Korean indigenous cattle) calves. Of the 140 fecal samples previously tested for *C. parvum*, which were obtained from Hanwoo calves aged 60 days, 70 tested positive and 70 tested negative. These samples were included in this study. By using the polymerase chain reaction (PCR) analysis targeting the *RdRp* gene of CSpV1, we detected CSpV1 in 28 samples (20.0%), with infection rates of 31.4% (22/70) in *C. parvum*-positive and 8.6% (6/70) in *C. parvum*-negative samples. CSpV1 samples detected in the same farm were clustered together. To the best of our knowledge, this is the first study to report the prevalence and molecular characteristics of CSpV1 in Hanwoo calves in the Republic of Korea, providing important insights into the relationship between *C. parvum* and CSpV1 in bovine hosts.

## 1. Introduction

*Cryptosporidium* spp. are intracellular coccidian parasites with a well-established enteric nature, primarily infecting a plethora of host species, most notably bovine species [1]. There were numerous species of *Cryptosporidium* spp. identified within cattle populations: *C. parvum*, *C. andersoni*, *C. bovis*, *C. ryanae*, *C. felis*, *C. hominis*, *C. meleagridis*, *and C. suis* [2]. Among them, *C*. *parvum*, *C*. *bovis*, *C*. *ryanae*, and *C*. *andersoni* have been revealed to be the primary causative agents for most bovine infections [3].

Neonatal calves infected with these pathogens often present with a spectrum of clinical symptoms. The most prominent among these include diarrhea, inappetence, and lethargy. If left unchecked, these symptoms can intensify, leading to dehydration and, in extreme cases, mortality [4]. The considerable morbidity and mortality associated with cryptosporidiosis have catalyzed numerous research initiatives aimed at mitigating its impact. Despite these efforts, the search for a definitive treatment protocol remains ongoing, and the prospects of an effective vaccine appear bleak [5,6]. Given these challenges, the emphasis has shifted towards a deeper understanding of host–parasite interactions, which are expected to underpin future preventive measures against this disease.

In the current absence of a dependable treatment or vaccine for various ailments, the diagnostic process assumes a paramount significance in disease management. The advent of *Cryptosporidium parvum* virus-1 (CSpV1) as a diagnostic tool presents a promising pathway. Initial investigations have indicated that diagnostic assays based on CSpV1 may exhibit exceptional sensitivity in comparison to conventional *Cryptosporidium* PCR that CSpV1 might be able to detected in a single oocyst of *Cryptosporidium* [7].

CSpV1, classified under the genus *Cryspovirus* within the *Partitiviridae* family, is a double-stranded RNA virus closely associated with *C*. *parvum* oocysts. Its genome is bifurcated into two segments. The dsRNA1 segment, with a size of 1.8 kbp, encodes the RNA-dependent RNA polymerase (RdRp), whereas the dsRNA2 segment, with a size of 1.5 kbp, is responsible for encoding the capsid protein [8]. Although the influence of CSpV1 on the virulence of *C*. *parvum* remains unclear, an emerging hypothesis suggests the potential of CSpV1 to enter a latent state within *C*. *parvum* post-infection. Recent research has hinted at a mechanism wherein *Cryptosporidium* might utilize CSpV1 to initiate type I IFN signaling, potentially circumventing the host’s epithelial antiparasitic defense [9,10].

Given these insights, the present study endeavors to ascertain the prevalence of CSpV1. Moreover, a detailed molecular characterization of CSpV1, particularly within Hanwoo (Korean indigenous cattle, *Bos taurus coreanae*) calves, forms an integral component of this research.

## 2. Materials and Methods

One hundred forty diarrheic fecal samples were selected from Hanwoo calves aged less than 60 days for CSpV1 detection. These samples were sent to the Animal Industry Data Korea Diagnostic Laboratories by on-field veterinary professionals between May and November 2022. Before performing the CSpV1 PCR, 70 samples were randomly selected from previously submitted fecal test results that were positive for *C. parvum*. For comparison, an equal number of *C. parvum*-negative samples from same farms, which had been submitted concurrently with the positive ones, were also chosen. Farms were located in four provinces in the Republic of Korea: four farms in Gyeonggi-do (Anseong), five farms in Chungcheongnam-do (Yesan, Dangjin, Gongju, and Cheongyang), three farms in Jeollabuk-do (Buan, Jeongeup, and Gimje), and one farm in Gyeongsangnam-do (Sancheong).

In our investigative procedure, the submitted fecal samples were suspended in 0.01 M phosphate-buffered saline, resulting in a 30% concentration of fecal homogenates. Subsequently, the samples underwent a brief centrifugation process for 1 min at a speed of 100× *g*. Total nucleic acids were extracted from the supernatant following the manufacturer’s instructions using the MagMAX™ Total Nucleic Acid Isolation Kit (Thermo Fisher Scientific, Waltham, MA, USA). All procedures were performed according to the manufacturer’s instructions. All extracts were stored at −70 °C until real-time polymerase chain reaction (PCR) was performed.

Real-time PCR to detect *C. parvum* was performed using the GoTaq One-Step RT-qPCR System (Promega, Madison, WI) following the manufacturer’s protocol with the following primers and probe: forward primer (5′-CAA ATT GAT ACC GTT TGT CCT TCT GT-3′), reverse primer (5′-GGC ATG TCG ATT CTA ATT CAG CT-3′), and probe (5′-JOE TGC CAT ACA TTG TTG TCC TGA CAA ATT GAA-BHQ1–3′) [11]. In a 10 µL reaction volume, 2 µL of the extracted template and 8 µL of the reaction mixture were used, with the latter consisting of 5 µL of GoTaq qPCR Master Mix 2X, 0.2 µL of GoScript™ RT Mix for 1-Step RT-qPCR 50X, 2 µL of primer and probe mixture, and 0.8 µL of RNase-Free dH_2_O. The final primer and probe concentrations were 0.3 µM and 0.2 µM, respectively. Real-time PCR was conducted using the CFX Opus 96 Real-Time PCR System (Applied Biosystems, Foster City, CA, USA), with the following cycling conditions: a 10 min activation step at 95 °C, followed by 40 cycles of 15 s at 95 °C and 60 s at 60 °C. Samples with a cycle threshold value of <35 were considered positive.

**Figure 1:**
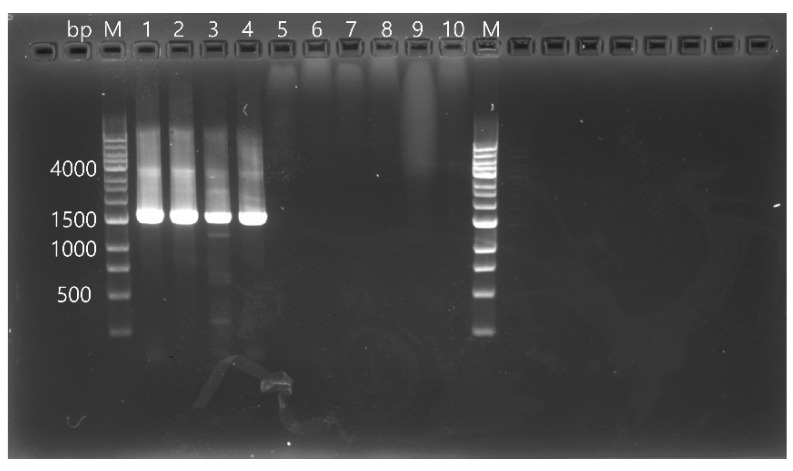
Representative agarose gel electrophoresis result of PCR targeting the *RdRp* gene of CSpV1. Amplified PCR products were separated by electrophoresis on a 1.0% agarose gel and visualized after staining with RedSafe™ nucleic acid staining solution. M: DNA ladder (1 kb); lanes 1–4: samples in which the *RdRp* gene of CSpV1 was detected (1576 bp); lanes 5–9: samples in which the *RdRp* gene of CSpV1 was not detected; lane 10: negative control.

To detect CSpV1, RT-PCR was performed using PrimeScript™ One-Step RT-PCR Kit Ver. 2 (Takara Bio Inc., Shiga, Japan) according to the manufacturer’s instructions, and all procedures were performed as previously described with the following primer set used for the amplification of the *RdRp* gene of CSpV1: forward primer (5′-AAG TTT GTC AAT ATC TAT GAG ATA C-3′) and reverse primer (5′-TCC ATA AAT TTT GTG ACT CCT G-3′) [12]. To detect CSpV1, total nucleic acids previously extracted and stored at −80 °C for *C*. *parvum* experiments were transferred to a 4 °C refrigerator prior to the experiments. Once fully thawed, all samples were immediately placed on ice before the PCR experiments were performed. A 25 µL reaction volume was prepared with 2 µL of the extracted template and 23 µL of the reaction mixture, which contained 1 µL of PrimeScript™ 1-step Enzyme Mix, 12.5 µL of 2 × 1-Step Buffer, 1 µL of primer mixture, and 8.5 µL of RNase-Free dH_2_O. The final primer concentration used was 0.4 µM. All PCR amplicons based on partial sequences of the *RdRp* gene of CSpV1 (1576 bp) were analyzed by gel electrophoresis on a 1.0% agarose gel stained with RedSafe™ nucleic acid staining solution (Intron Biotechnology Inc., Seongnam, Republic of Korea) (Figure 1) and sequenced directly using dideoxy termination with an automatic sequencer (3730xl Capillary DNA Analyzer; Applied Biosystems, Foster City, CA, USA). The obtained sequences were applied to a Basic Local Alignment Search Tool (BLAST) in the National Center for Biotechnology Information (NCBI) database. The sequences were aligned using Clustal X (Ver 2.0) and examined with a similarity matrix. Phylogenetic analysis was performed using the neighbor-joining method based on nucleotide alignments. Bootstrap analysis was conducted with 1000 replicates using the MEGA version X.

All statistical methods were performed in SPSS (Version 25.0; IBM Corp., Armonk, NY, USA). The PCR results of the fecal samples were recorded as positive and negative for *C*. *parvum* and CSpV1 and the association between the two pathogens was assessed using the Pearson’s χ^2^ test, accompanied by the calculation of odds ratios and 95% confidence intervals.

## 3. Results

In the present study, we analyzed a total of 140 fecal samples obtained from 13 distinct Hanwoo farms. A comprehensive breakdown of the samples and the results of CSpV1 detection is described in Table 1. Out of these 140 samples, 28 (20.0%) tested positive for CSpV1. These positive samples originated from seven farms dispersed across four provinces in the Republic of Korea. Specifically, there were two farms in Jeollabuk-do (Buan (Farm 1) and Jeongeup (Farm 3)), four in Chungcheongnam-do (Dangjin (Farm 2), Cheongyang (Farms 4 and 5), and Gongju (Farm 6)), and one in Gyeonggi-do (Anseong (Farm 7)). We noted that the prevalence of CSpV1 in the *C*. *parvum*-positive samples stood at 31.4% (22 out of 70), whereas it was 8.6% (6 out of 70) in the *C. parvum*-negative samples. The difference was statistically significant (*p* < 0.01) with an odds ratio of 4.889 and a 95% confidence interval ranging from 1.84 to 12.99. Breaking down the CSpV1-positive samples by farm and their *C. parvum* status revealed the following: On Farm 1, out of four CSpV1-positive samples, three tested positive for *C*. *parvum*, while one tested negative. Farm 5 had a total of eight CSpV1-positive samples, with seven of them being *C*. *parvum*-positive and just one being negative. Farm 7’s two CSpV1-positive samples both tested positive for *C*. *parvum*. Similarly, Farm 8’s one CSpV1-positive sample was *C*. *parvum*-positive. All three CSpV1-positive samples from Farm 9 were confirmed as *C*. *parvum*-positive. Lastly, of the nine CSpV1-positive samples from Farm 10, five were *C*. *parvum*-positive and the remaining four were negative (Table 1).

**Figure 2:**
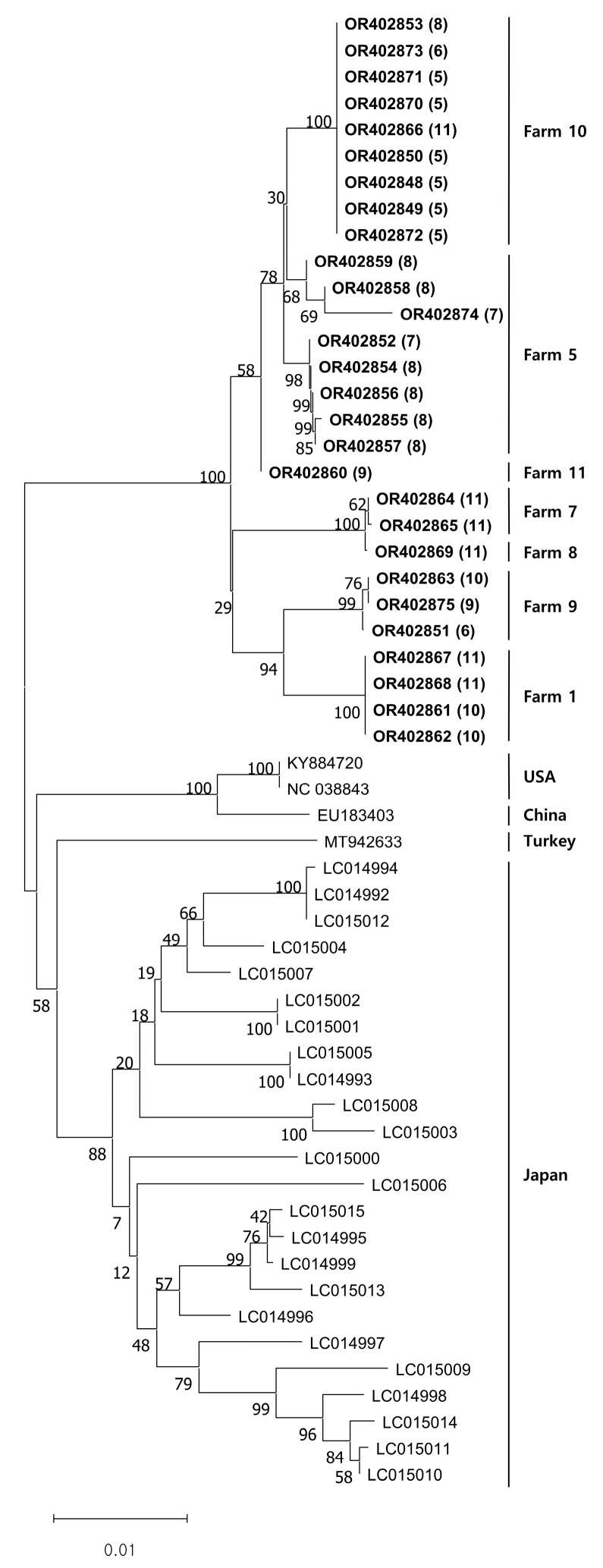
Phylogenetic analysis of *Cryptosporidium parvum* virus-1 (CSpV1). Neighbor-joining phylogenetic trees were based on partial sequences of the CSpV1 *RdRp* sequences (1576 bp). The bar represents genetic distance. Numbers at nodes indicate bootstrap percentages obtained after 1000 bootstrap replicates. The obtained sequences in this study were marked with bold letters. Sampling months in 2022 are indicated by a () after the GenBank number.

All the viral DNA sequences we obtained were registered under accession numbers OR402848 to OR402875. The DNA sequence similarity was 98.3–100% among our sequences. When compared to sequences from other countries, the similarity ranged from 95.6% to 96.5% for sequences from the USA, 95.5% to 96.4% for sequences from China, 95.4% to 96.2% for sequences from Turkey, and 94.6% to 96.8% for sequences from Japan. Our phylogenetic analysis indicated that the CSpV1 isolates clustered according to their source farms. Additionally, previously documented foreign CSpV1 sequences also appeared to cluster by their respective nations (Figure 2).

## 4. Discussion

This research was conducted to identify the prevalence and molecular characterization of CSpV1 and their relationship with *C. parvum* in 140 Hanwoo calves. All calves in this study were less than 60 days of age. However, the exact age of several samples was not recorded at the time of submission, making it impossible to calculate the average age of the calves.

In this study, CSpV1 was detected in 28 out of 140 samples, representing a 20.0% prevalence. Among the 70 samples that were positive for *C. parvum*, 22 (31.4%) also tested positive for CSpV1. This rate was statistically significantly higher than in the *C. parvum*-negative samples (*p* < 0.01). No statistical correlation was observed between individual farms and CSpV1 infection (results not shown). Interestingly, the detection rate of CSpV1 in *C*. *parvum*-positive feces was found to be lower than that observed in a previous study [13]. It can be hypothesized that this might be attributed to the DNA/RNA of feces already being extracted for the previous detection of *C*. *parvum* and stored for nearly a year. Furthermore, the sensitivity and specificity may vary based on the method utilized. It is worth noting that previous diagnostic methods for CSpV1 typically included purifying *Cryptosporidium* oocysts [12,13]. Nevertheless, this study successfully detected CSpV1 via direct nucleic acid extraction from fecal samples and PCR. This is especially noteworthy as purifying *Cryptosporidium* oocysts from the feces of hosts is an arduous and time-consuming process. The successful detection of CSpV1 in clinical samples could potentially be a promising method for tracking *Cryptosporidium*. However, it is important to note that an improvement in the sensitivity of detection methods is necessary.

Genomic studies of *Cryptosporidium* oocysts pose unique obstacles that differentiate them from standard DNA extraction methods used for bacteria or viruses, making CSpV1 a promising tool for monitoring *Cryptosporidium*. One significant challenge is the study exterior of the oocyst, which shows greater resilience than bacterial cell walls or viral capsids and requires specialized lysis techniques. The oocyst wall’s complicated composition, which includes filamentous glycoproteins (oocyst wall proteins or OWPs) and acid-fast lipids, complicates DNA extraction using conventional methods [14,15]. As a result, CSpV1 has the potential to overcome these issues and to provide accurate genomic data. This tool’s efficiency in tracking *Cryptosporidium* could advance our understanding of the parasite’s epidemiology and improve disease control measures. CSpV1 has the potential to be a valuable asset for researchers studying *Cryptosporidium* and to contribute to the development of effective preventative measures.

Phylogenetic analysis in this study revealed that the *RdRp* gene of CSpV1 was grouped into the same cluster (Figure 1). Intriguingly, an overwhelming majority of CSpV1 strains that were discovered on this specific farm consistently belonged to the same cluster. Clusters within the analysis demonstrated a consistent pattern, forming regardless of whether the results for *C. parvum* were positive or negative, and this trend was consistently observed across various months when samples were collected, as clearly illustrated in Figure 1. Furthermore, considering the gene sequences of *RdRp* of CSpV1 from various countries, they all seemed to align and to be categorized into that identical cluster. This observation and these data strongly align with previous findings, where the *RdRp* genes of CSpV1 in Japan were found to be clustering in a specific manner, based on the geographical distribution of their respective hosts [13].

In this research, since real-time PCR was utilized specifically for detecting *C*. *parvum*, we did not perform *C*. *parvum* subtyping. Nevertheless, there were other reports about the relationship between *C*. *parvum* and CSpV1. Previous research in Japan pinpointed the presence of CSpV1 within the subtype IIaA15G2R1 of *C*. *parvum* [13]. Expanding on this knowledge, recent studies from France have uncovered CSpV1 within multiple *C*. *parvum* subtypes, including IIaA15G2R1, IIaA17G2R1, and IIdA18G1R1 [15]. An observation of particular note is the geographical clustering pattern of CSpV1, which appears to be consistent regardless of the specific subtype of *C*. *parvum* with which it is associated. Considering an earlier study that emphasized the notable variability within the RdRp gene, as well as its unique, species-specific affiliation with its respective host, it seems both appropriate and essential to intensify research efforts to better understand the intricate relationship between CSpV1 and *C*. *parvum* [12].

While this study focused on the relationship between *C. parvum* and CSpV1, further research is necessary as CSpV1 has also been found in other *Cryptosporidium* species, including *C. bovis*, *C. ryanae*, *C. felis*, *C. hominis*, *and C. meleagridis* [7,16]. In this study, six CSpV1 isolates were detected in 70 *C. parvum*-negative feces samples, suggesting infection with *Cryptosporidium* spp. other than *C. parvum*. To date, *C. parvum*, *C*. *andersoni*, *C. bovis*, and *C*. *ryanae* have been reported as *Cryptosporidium* spp. associated with calf diarrhea in the Republic of Korea, and CSpV1 in other species of *Cryptosporidium* has been reported in other countries [7,17]. Furthermore, although it has not been experimentally verified in this study, previous reports suggested that CSpV1 forms the same cluster with different subtypes of *C*. *parvum*, suggesting a high occurrence of host shift for CSpV1 [18]. In light of this, it can be postulated that CSpV1 might not be restricted to *C*. *parvum* and could potentially exhibit a host shift even in infections caused by other species of *Cryptosporidium* spp. since CSpV1 in *C*. *parvum* negative samples in this research was grouped in the same cluster as others. Therefore, further research is needed to identify the relationship between CSpV1 and *Cryptosporidium* spp.

*Cryptosporidium* has been identified as a principal etiological agent of diarrhea in calves, infiltrating and compromising the mucosal lining of the small intestine. This action precipitates a notable reduction in villi functionality and an augmentation in crypt regions, thereby disrupting the physiological mechanisms of digestion and nutrient absorption, a sequence culminating in diarrhea hallmarked by significant nutrient malabsorption [19]. During the advancement of the disease, calves exhibit a series of clinical manifestations encompassing severe diarrhea, pronounced lethargy, acute dehydration, diminished feed intake, and abdominal discomfort, with a heightened risk of tenesmus being observed [20].

Globally, and notably in Korea, *Cryptosporidium* species have been highlighted as a prevalent causative agent of calf diarrhea. Korean studies have documented an infection rate pegged at 9.9% in cases of calf diarrhea, a figure that further underscores the significance of this pathogen in veterinary medicine [21]. In a broader context, large-scale investigations in the United States have recorded an infection rate of 43.0% in heifer calves, an alarming statistic that echoes the global concern over this parasite’s impact on bovine health [22].

Despite the substantial morbidity associated with this parasitic infection, the diagnostic process remains a challenging endeavor, with effective preventative and therapeutic interventions still in the developmental phase. It is projected that a holistic approach towards research, encompassing not only the exploration of associations between CSpV1 and *Cryptosporidium* in the diagnostic sphere but also delving into their pathogenic mechanisms, could potentially position CSpV1 as a viable alternative solution to mitigating the detrimental effects of *Cryptosporidium* infections in calves, thus fostering advancements in veterinary science.

## 5. Conclusions

In conclusion, this study compared the infection rates of CSpV1 between equal numbers of *C*. *parvum*-positive and *C*. *parvum*-negative fecal samples from 140 Hanwoo calves. We assessed the prevalence and molecular characterization of the detected CSpV1. It was observed that the CSpV1 isolates formed clusters based on geographical characteristics, irrespective of the presence or absence of *C*. *parvum* infection and the month of sampling. To the best of our knowledge, this represents the first study identifying the prevalence and molecular characteristics of CSpV1 associated with calf diarrhea in the Republic of Korea.

## Figures and Tables

**Table 1:** The description of submitted feces and CSpV1 PCR results from 140 Hanwoo calves in 13 farms.

Farms	Province	Location	Number of Samples *	No. of CSpV1-Positive Samples (CSpV1 Positivity among *C. parvum* Results for Each Farm and Location, %)		
				*C*. *parvum* Positive	*C*. *parvum* Negative	Total
Farm 1	Gyeonggi-do	Anseong	14	3 (42.9)	1 (14.3)	4 (28.6)
Farm 2		Anseong	6	0 (0.0)	0 (0.0)	0 (0.0)
Farm 3		Anseong	42	0 (0.0)	0 (0.0)	0 (0.0)
Farm 4		Anseong	2	0 (0.0)	0 (0.0)	0 (0.0)
Subtotal			64	3 (9.4)	1 (3.1)	4 (6.3)
Farm 5	Chungcheongnam-do	Dangjin	28	7 (50.0)	1 (7.1)	8 (28.6)
Farm 6		Yesan	4	0 (0.0)	0 (0.0)	0 (0.0)
Farm 7		Cheongyang	6	2 (66.7)	0 (0.0)	2 (33.3)
Farm 8		Cheongyang	2	1 (100.0)	0 (0.0)	1 (50.0)
Farm 9		Gongju	8	3 (75.0)	0 (0.0)	3 (37.5)
Subtotal			48	13 (54.2)	1 (4.2)	14 (29.2)
Farm 10	Jeollabuk-do	Buan	18	5 (55.6)	4 (44.4)	9 (50.0)
Farm 11		Jeongeup	2	1 (100.0)	0 (0.0)	1 (50.0)
Farm 12		Gimje	4	0 (0.0)	0 (0.0)	0 (0.0)
Subtotal			24	6 (50.0)	4 (33.3)	10 (41.7)
Farm 13	Gyeongsangnam-do	Sancheong	4	0 (0.0)	0 (0.0)	0 (0.0)
Subtotal			4	0 (0.0)	0 (0.0)	0 (0.0)
Total			140	22 (31.4)	6 (8.6)	28 (20.0)

* Each farm had an equal number of *C. parvum*-positive and *C. parvum*-negative samples.

## Data Availability

The data that support the findings of this study are available from the corresponding author upon reasonable request.

## References

[1] Ryan U., Fayer R., Xiao L. (2014). *Cryptosporidium* species in humans and animals: Current understanding and research needs. Parasitology.

[2] Dărăbuș G., Lupu M.A., Mederle N., Dărăbuș R.G., Imre K., Mederle O., Imre M., Paduraru A.A., Morariu S., Olariu T.R. (2023). Epidemiology of *Cryptosporidium* infection in Romania: A Review. Microorganisms.

[3] Lichtmannsperger K., Harl J., Freudenthaler K., Hinney B., Wittek T., Joachim A. (2020). *Cryptosporidium parvum*, *Cryptosporidium ryanae*, and *Cryptosporidium bovis* in samples from calves in Austria. Parasitol. Res..

[4] Santin M. (2020). *Cryptosporidium* and *Giardia* in ruminants. Vet. Clin. N. Am. Food Anim. Pract..

[5] Ryan U., Zahedi A., Paparini A. (2016). *Cryptosporidium* in humans and animals-a one health approach to prophylaxis. Parasite Immunol..

[6] Sparks H., Nair G., Castellanos-Gonzalez A., White A.C. (2015). Treatment of *Cryptosporidium*: What we know, gaps, and the way forward. Curr. Trop. Med. Rep..

[7] Shehata A.A., Bando H., Fukuda Y., Kabir M.H.B., Murakoshi F., Itoh M., Fujikura A., Okawa H., Takuto E., Akira G. (2020). Development of a highly sensitive method for the detection of *Cryptosporidium parvum* virus type 1 (CSpV1). Jpn. J. Vet. Res..

[8] Gallimore C.I., Green J., Casemore D.P., Brown D.W. (1995). Detection of a Picobirnavirus associated with *Cryptosporidium* positive stools from humans. Arch. Virol..

[9] Deng S., He W., Gong A.Y., Li M., Wang Y., Xia Z., Zhang X.T., Huang Pacheco A.S., Naqib A., Jenkins M. (2023). *Cryptosporidium* uses CSpV1 to activate host type I interferon and attenuate antiparasitic defenses. Nat. Commun..

[10] Vainio E.J., Chiba S., Ghabrial S.A., Maiss E., Roossinck M., Sabanadzovic S., Suzuki N., Xie J., Nibert M., Ictv Report Consortium (2018). ICTV virus taxonomy profile: Partitiviridae. J. Gen. Virol..

[11] Cho Y.I., Han J.I., Wang C., Cooper V., Schwartz K., Engelken T., Yoon K.J. (2013). Case–control study of microbiological etiology associated with calf diarrhea. Vet. Microbiol..

[12] Berber E., Şimşek E., Çanakoğlu N., Sürsal N., Gençay Göksu A. (2021). Newly identified *Cryptosporidium parvum* virus-1 from newborn calf diarrhoea in Turkey. Transbound. Emerg. Dis..

[13] Murakoshi F., Ichikawa-Seki M., Aita J., Yaita S., Kinami A., Fujimoto K., Nishikawa Y., Murakami S., Horimoto T., Kato K. (2016). Molecular epidemiological analyses of *Cryptosporidium parvum* virus 1 (CSpV1), a symbiotic virus of *Cryptosporidium parvum*, in Japan. Virus Res..

[14] Claudel L., Valeix N., Basmaciyan L., Pereira B., Costa D., Vincent A., Valot S., Favennec L., Dalle F. (2021). Comparative study of eleven mechanical pretreatment protocols for *Cryptosporidium parvum* DNA extraction from stool samples. Microorganisms.

[15] Valeix N., Costa D., Basmaciyan L., Valot S., Vincent A., Razakandrainibe R., Robert-Gangneux F., Nourrisson C., Pereira B., Fréalle E. (2020). Multicenter comparative study of six *Cryptosporidium parvum* DNA extraction protocols including mechanical pretreatment from stool samples. Microorganisms.

[16] Leoni F., Gallimore C.I., Green J., McLauchlin J. (2003). Molecular epidemiological analysis of *Cryptosporidium* isolates from humans and animals by using a heteroduplex mobility assay and nucleic acid sequencing based on a small double-stranded RNA element. J. Clin. Microbiol..

[17] Jang D.H., Cho H.C., Park Y.J., Park J., Choi K.S. (2023). First report of *Cryptosporidium andersoni* and risk factors associated with the occurrence of *Cryptosporidium* spp. in pre-weaned native Korean calves with diarrhea. Front. Vet. Sci..

[18] Adjou K.T., Chevillot A., Lucas P., Blanchard Y., Louifi H., Arab R., Mammeri M., Thomas M., Polack B., Karadjian G. (2023). First identification of Cryptosporidium parvum virus 1 (CSpV1) in various subtypes of Cryptosporidium parvum from diarrheic calves, lambs and goat kids from France. Vet. Res..

[19] Di Genova B.M., Tonelli R.R. (2016). Infection Strategies of Intestinal Parasite Pathogens and Host Cell Responses. Front. Microbiol..

[20] Adkins P.R.F. (2022). Cryptosporidiosis. Vet. Clin. N. Am. Food Anim. Pract..

[21] Lee S.H., Van Bik D., Kim H.Y., Lee Y.R., Kim J.W., Chae M., Oh S.I., Goo Y.K., Kwon O.D., Kwak D. (2016). Multilocus typing of Cryptosporidium spp. in young calves with diarrhea in Korea. Vet. Parasitol..

[22] Urie N.J., Lombard J.E., Shivley C.B., Kopral C.A., Adams A.E., Earleywine T.J., Olson J.D., Garry F.B. (2018). Preweaned heifer management on US dairy operations: Part I. Descriptive characteristics of preweaned heifer raising practices. J. Dairy Sci..

